# Porewater methane transport within the gas vesicles of diurnally migrating *Chaoborus* spp.: An energetic advantage

**DOI:** 10.1038/srep44478

**Published:** 2017-03-14

**Authors:** Daniel F. McGinnis, Sabine Flury, Kam W. Tang, Hans-Peter Grossart

**Affiliations:** 1 Aquatic Physics Group, Department F.-A. Forel for Environmental and Aquatic Sciences (DEFSE), Section of Earth and Environmental Sciences, Faculty of Sciences, University of Geneva, Uni Carl Vogt, 66 boulevard Carl-Vogt, CH-1211 Geneva, Switzerland; 2 Leibniz-Institute of Freshwater Ecology and Inland Fisheries (IGB), Mueggelseedamm 310, 12587 Berlin, Germany; 3 Department of Biosciences, Swansea University, Wallace Building 104, SA2 8PP Swansea, UK; 4 Potsdam University, Institute of Biochemistry and Biology, Maulbeerallee 2, 14469 Potsdam, Germany

## Abstract

Diurnally-migrating *Chaoborus* spp. reach populations of up to 130,000 individuals m^−2^ in lakes up to 70 meters deep on all continents except Antarctica. Linked to eutrophication, migrating *Chaoborus* spp. dwell in the anoxic sediment during daytime and feed in the oxic surface layer at night. Our experiments show that by burrowing into the sediment, *Chaoborus* spp. utilize the high dissolved gas partial pressure of sediment methane to inflate their tracheal sacs. This mechanism provides a significant energetic advantage that allows the larvae to migrate via passive buoyancy rather than more energy-costly swimming. The *Chaoborus* spp. larvae, in addition to potentially releasing sediment methane bubbles twice a day by entering and leaving the sediment, also transport porewater methane within their gas vesicles into the water column, resulting in a flux of 0.01–2 mol m^−2^ yr^−1^ depending on population density and water depth. *Chaoborus* spp. emerging annually as flies also result in 0.1–6 mol m^−2^ yr^−1^ of carbon export from the system. Finding the tipping point in lake eutrophication enabling this methane-powered migration mechanism is crucial for ultimately reconstructing the geographical expansion of *Chaoborus* spp., and the corresponding shifts in the lake’s biogeochemistry, carbon cycling and food web structure.

*Chaoborus* spp., or phantom-midge larvae, are widely distributed in both temperate and tropical lakes^1^ ^and references therein^ with depths up to 70 meters^2^. As *Chaoborus* spp. can be present in large quantities in lakes (ca. 2,000–130,000 individuals m^−2^), they can have significant impacts on lake ecology^3^, and their appearance in the lake’s history may signal a “tipping point” in trophic status (Fig. 1).

Though generally unaccounted for in lake studies and models, *Chaoborus* spp. are often the most important invertebrate predators^4^ and food source for fish^5^, bioturbate sediments^3^ (including methane bubble release^6^), are a significant oxygen sink through respiration^7^, and represent a carbon loss upon emergence and thus an important link to the terrestrial ecosystem^8^. *Chaoborus* spp. are commonly found in eutrophic and dystrophic lakes, yet are absent in oligotrophic lakes^9^. Consequently, they are often used as paleolimnological indicators for reconstructing eutrophication history^10^. Linked to climate change, *Chaoborus* spp. have been recently shown to expand their habitat across tundra thaw ponds^11^.

Spending most of their lives in the larval stage in lakes (1–2 years), the third- and fourth-instar of some *Chaoborus* spp. larvae (e.g. *C. flavicans*, *C. punctipennis*, *C. trivitattus*) diurnally migrate in the presence of fish^1^,^12^,^13^,^14^: dwelling in the sediment during daytime and feeding at night in the surface layer. *Gosselin and Hare*^3^ observed them migrating into and out of the anoxic sediment, which disturbs and enhances exchange across the top sediment layer. This bioturbation has been shown to result in the release of a large amount of gas bubbles from the sediment during emergence of *Chaoborus* spp. larvae from the sediments of a Brazilian reservoir^6^. In the sediment, *Chaoborus* spp. larvae burrow beneath the oxic zones several centimeters deep and position themselves in a vertical “S” shape^3^. In addition to predator avoidance, this migration behavior helps the larvae to reduce their metabolic expenditure by staying in the often-colder hypolimnion^7^. The conservation of energy is critical, as lower energy requirements allow migrating *Chaoborus* spp. to expand their habitat to deeper and less productive (less prey) lakes.

*Chaoborus* spp. adjust or correct their position in the water column using their two sets of gas-filled, semi-rigid tracheal sacs (gas sacs) which they can adjust by ca. ± 20–25% volume^15^ ^and references therein^; however, it is not known whether they also use these sacs to aid diurnal migration^7^,^16^. Migrating *Chaoborus* spp. are found in lakes no deeper than 70 m^2^, and at depths greater than 100 m their tracheal respiration system would collapse^17^. *Nilssen*^18^ reported that only *C. flavicans* occurred at depths greater than 5 m in Norwegian Lakes (down to 60 m), suggesting other species (i.e. *C. obscuripes* and *C. crystallinus*) cannot withstand higher hydrostatic pressure due to the morphology of their gas sacs.

*Chaoborus* spp. control gas diffusion across the sac wall with a protein that expands or contracts based on pH^15^,^17^,^19^. The expansion of the tracheal sac is suggested to be due to changes in the sac wall plasticity, coupled with an “extending force” – i.e. extrinsic muscles, an intimal component, or gas pressure^15^. To our knowledge, no explanation has yet been presented as to how (or if) these insects are able to expand their sacs at depth against high hydrostatic pressure.

As *Chaoborus* spp. mandibles are well-preserved in the sediment, they are often used as paleolimnological indicators of eutrophication history of lakes^10^,^20^,^21^. Many studies have attempted to search for reliable eutrophication-related predictors of *Chaoborus* spp. presence. Oxygen levels seem to be an obvious choice, as it has been suggested that *Chaoborus* spp. utilize anoxic regions to avoid predation^20^. They are tolerant of hydrogen sulfide (H_2_S) and are able to respire anaerobically using a malate cycle to produce ATP^3^,^17^ ^and references therein^. However, correlations of *Chaoborus* spp. presence with hypolimnetic oxygen levels are sometimes weak or even contradictory^10^, suggesting additional unknown factors are needed to explain their presence. One of these factors may be methane, since it has the potential to affect the buoyancy mechanism and hence the energetic expenditure and habitat range of migrating *Chaoborus* spp.

We suggest that *Chaoborus* spp. utilize the high partial-pressure of dissolved gases in sediment porewater as a transport mechanism to allow them to migrate via buoyancy (Fig. 1). The likely source of saturated or over-saturated gas partial pressure would be high levels of dissolved methane (CH_4_) in the porewater resulting from methanogenesis^22^. Carbon dioxide (CO_2_) and H_2_S, while present, are about 27 and 80 times more soluble than CH_4_, respectively, thus providing insignificant partial pressures for gas sac expansion^23^. Combined with dissolved nitrogen (N_2_), the other most abundant sparingly-soluble gas in the sediment porewater, the two gases (CH_4_ and N_2_) lead to high enough partial pressures (matching or exceeding the ambient hydrostatic pressure) to effortlessly inflate the *Chaoborus* spp. gas sacs when in contact with the porewater. This provides an energetic advantage for migration through passive buoyancy rather than active swimming. This unique adaptation has the potential to greatly reduce metabolic expenditure, and subsequently allow *Chaoborus* spp. to thrive in deeper environments and in those with lower food availability. As important side-effects, migrating *Chaoborus* spp. transport porewater methane to the water column directly in their gas sacs and bioturbate methane-rich sediments, allowing methane to bypass diffusive and oxidation limitations at the sediment-water interface.

It is critical to understand limnological conditions and variables which promote lakes as suitable habitats for *Chaoborus spp*. In this paper, we explore the energetics and methane gas transport involved in this proposed migratory adaptation by: (1) studying the methane gas transfer into and out of the gas sacs; (2) investigating the physics of the *Chaoborus’* rise and descent; (3) comparing the energetic requirements for swimming vs. buoyant locomotion; and (4) investigating the effect on methane fluxes from the sediment to the overlying water column.

## Results

Our experiments confirmed that *Chaoborus* spp. initially took up the dissolved methane when exposed to methane-saturated water (~1.5 mmol L^−1^). After the initial exposure to the methane-saturated water and subsequent transferal to the experimental “methane-free” flask (~3 nmol L^−1^), methane in their gas sacs was redissolved into the surrounding water and into the head space as indicated by the increase in head-space CH_4_ concentration measured by the gas analyzer (Fig. 2a).

In the first experiment, 35 methane pre-treated larvae were transferred to the experimental flask, and changes in headspace gas concentrations were monitored continuously. The headspace CH_4_ concentration gradually increased until it reached a steady state at 110 minutes (Fig. 2a). These results are consistent with the experiments of *Teraguchi*^15^, who showed that the gas concentration in the gas sacs approached equilibrium with the surrounding dissolved gas concentrations (enriched argon, etc.). In the second experiment, the steady state increase was 3.29, 6.46 and 8.90 ppm CH_4_ for 13, 25 and 35 individuals, respectively. Therefore, the CH_4_ increase was proportional to the number of *C. flavicans* in the flask (0.255 ± 0.003 ppm CH_4_ ind.^−1^), and was close to nil in the control flask (Fig. 2b).

Stoke’s rise velocity: We calculate the theoretical rising (and sinking) velocity of a single *Chaoborus* larvae based on an adjustment of its total gas sac volume by ± 25%^15^. In these calculations, we assume a homogeneous water column and that neutral density is achieved at ρ = 1 mg mm^−3^. A *Chaoborus* larva of 1 mm in diameter and 11 mm in length^3^ has a volume of 8.6 mm^3^. Expanding or contracting its gas sac volume of 0.12 mm^3^ by ± 25% will change its density by ± 0.35%. Using these values in equation 2 (see methods) gives a rising (or sinking) speed of 4.1 mm s^−1^. This is in good agreement with the migration speed of ~5 mm s^−1^ measured acoustically by Lorke *et al*.^13^.

## Discussion

Bioenergetics of migration: Several studies have related *Chaoborus* spp. presence to eutrophication of lakes^10^,^20^,^24^,^25^, but to our knowledge no explanation has been provided for this relationship based on the larvae’s migration energetics. *Giguere*^16^ experimentally measured the energy required for swimming at 0.07 mJ motion^−1^ with an average horizontal displacement of 2.5 cm motion^−1^ (2.8 mJ m^−1^). A simple analysis is performed assuming a resting metabolism of 490 mJ d^−1^ at 10 °C^7^,^16^ and a constant lake temperature. With these assumptions, the increase in energy expenditure for migration by active swimming (roundtrip) is shown in Fig. 3: A *Chaoborus* larva migrating between 45 m and the surface (i.e. 90 m total per day) would increase its energy demand by 50% see ref. 16 and nearly 80% in 70 m water depth.

To put this energy expenditure into perspective: A functional response study showed that a fourth instar *Chaoborus* has a maximum ingestion rate of 5 *Daphnia pulex* (1.44 mm size) per day^26^. Assuming an energy content of 185 mJ ind^−1^ for *Daphnia*^27^, this translates to a maximum energy intake of 925 mJ d^−1^ for the *Chaoborus* larva. Thus, diurnal migration through a 45–70 m water column by swimming would use up 80–95% of the larva’s maximum energy intake, and considerably more in warmer or less productive waters. Therefore, there is a clear and substantial energetic advantage for *Chaoborus* spp. to exploit the buoyancy provided by their methane-filled gas sacs for migration.

While *Chaoborus* spp. are commonly found in lakes all over the world, there are no comparable examples in the open ocean despite the vast abundance and diversity of insects^17^. This gas-supersaturation buoyancy mechanism would not work in ocean settings, as methane production occurs much deeper in marine sediments beneath the sulfate reduction zone^28^. Because CO_2_ and H_2_S are much more soluble than CH_4_ (~27 and 80 times respectively)^23^, the total dissolved gases in the porewater (including dissolved N_2_) of surficial marine sediments likely do not approach the supersaturated concentrations needed to inflate gas sacs.

The process of *Chaoborus* spp. inflating their gas sacs occurs very naturally and passively by the larvae being in contact with the porewater with saturated to oversaturated gas pressures. The only variable is the larvae’s adjustment of the gas diffusion rate across the gas sac wall^15^. Therefore, we suggest porewater methane as a potential but hitherto overlooked factor of the prevalence of migrating *Chaoborus* spp. in lakes. This is crucial to understanding their past and present distributions in lakes, and their impacts on carbon dynamics in lakes and in the surrounding landscapes, which we discuss below.

The work of *Teraguchi*^15^ and our results both demonstrate that the gases making up the sac contents are a product of sparingly-soluble gases in the surrounding environment. For *Chaoborus* spp. dwelling in lake sediment, the gas in their gas sacs consists largely of methane, which is re-dissolved in the water column during upward migration. Assuming *Chaoborus* spp. densities of 2,000 to 130,000 ind. m^−2^ 3 and a sac volume of 0.12 μL^15^, the migration-driven methane flux from the sediment to the water column would be ca. 10–2,000 mmol m^−2^ yr^−1^ for depths ranging from 20–70 m. This flux approaches and even exceeds typical diffusive fluxes across the sediment-water interface^29^,^30^, and is then dissolved throughout the water column as the *Chaoborus spp*. migrate from the sediment towards the surface layer to feed. The proportion dissolved in the hypolimnion and surface layer would depend on the lake depth and will be investigated in future studies.

Additionally, hydroacoustic surveys show that bioturbation by migrating *Chaoborus* spp. larvae at the surface sediment leads to increased gas bubble release from the sediment^6^. We therefore speculate that *Chaoborus* spp. larvae greatly affect methane transport from the lake sediment to the water column and atmosphere and could, in some cases, be a partial source of the often observed mid-water methane peak^31^. Furthermore, it illustrates the potential for other volatile and sparingly-soluble chemicals to be transported across the sediment-water interface via *Chaoborus* gas sacs and bioturbation.

In lakes with a history of eutrophication and pollution, the sediment can act as a reservoir of nutrients and pollutants, and diffusive release of solutes from sediment porewater can sustain and exacerbate internal chemical loading within the water column. While bubbles in cohesive sediment due to methane actually inhibit diffusion of other solutes to the water^32^, it is suggested that *Chaoborus* spp. can, through bioturbation, significantly enhance solute flux from sediment to the water column and subsequently increase lake internal loading^3^. *Gosselin and Hare*^3^ estimate that it takes between 9.3 days (2,300 ind m^−2^) and 0.2 days (130,000 ind m^−2^) to turnover 1 m^2^ of sediment by *Chaoborus*. Expressed as an apparent diffusivity, 2,000 ind m^−2^ enhance the surface sediment diffusivity 1,000 times over molecular diffusion, a substantial increase. In fact, *Chaoborus* spp. bioturbation is strong enough to even destroy the calcareous laminations in sediment deposits^33^.

*Chaoborus* spp. emergence represents a potentially significant carbon export from the system, and a link between the sediment biogeochemistry and the surrounding terrestrial ecosystem^34^. Estimates for adult *Chaoborus* spp. dry weight range from 1–1.8 mg ind^−1^ 16,35. Assuming 40% carbon and a mean dry weight of 1.4 mg ind^−1^, *Chaoborus* spp. emergence once per year gives a carbon export ranging from 0.1–6 mol m^−2^ yr^−1^ (1–72 g C m^−2^ yr^−1^) for densities of 2,300 to 130,000 ind m^−2^. Extrapolating this to the total lake surface area (4% of land area^36^) yields a global flux of ca. 7–400 Tg C yr^−1^. This is a very simple estimate as C*haoborus* spp. are absent in oligotrophic and deep (>70 m) lakes, yet it does not include small ponds. In addition to the carbon flux, emergent insects can be important links between sediment pollutants and surrounding predators (e.g. spiders)^34^.

**Figure 1:**
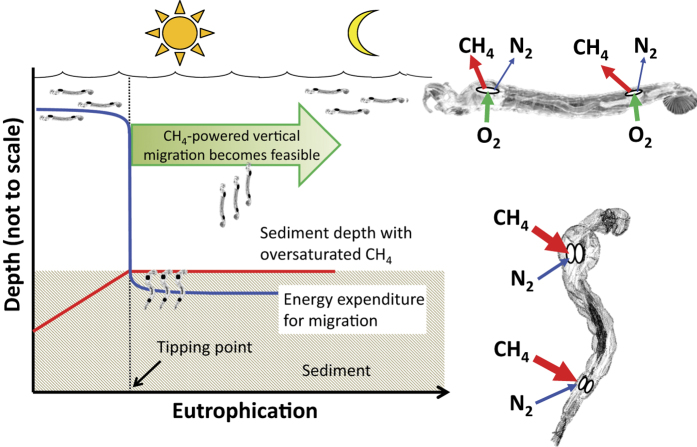
Conceptual diagram illustrating the tipping point in the eutrophication history of a lake when surface sediment methane concentration (red line) becomes sufficiently high to support methane-powered vertical migration of *Chaoborus* spp. This allows *Chaoborus* spp. to expand their habitable range and perform diurnal vertical migration with minimal energy expenditure (blue line). The right inserts show gaseous exchange between gas sacs and ambient water when the *Chaoborus* spp. reside in the sediment (bottom) vs. in the water column (top).

**Figure 2:**
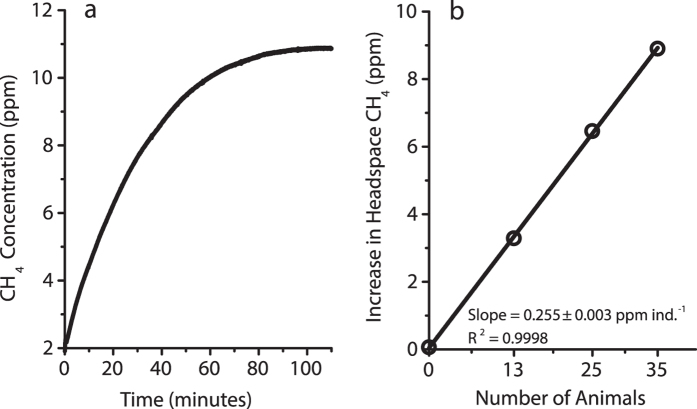
(**a**) Increase in headspace CH_4_ concentration over 100 minutes in the experimental flask with 35 animals and (**b**) results from 13, 25 and 35 methane pre-treated *Chaoborus* at equilibrium after 60 min. Control flask (at 0 on the axis) in (**b**) contained 25 *Chaoborus* pre-treated with CH_4_-free lake water.

**Figure 3:**
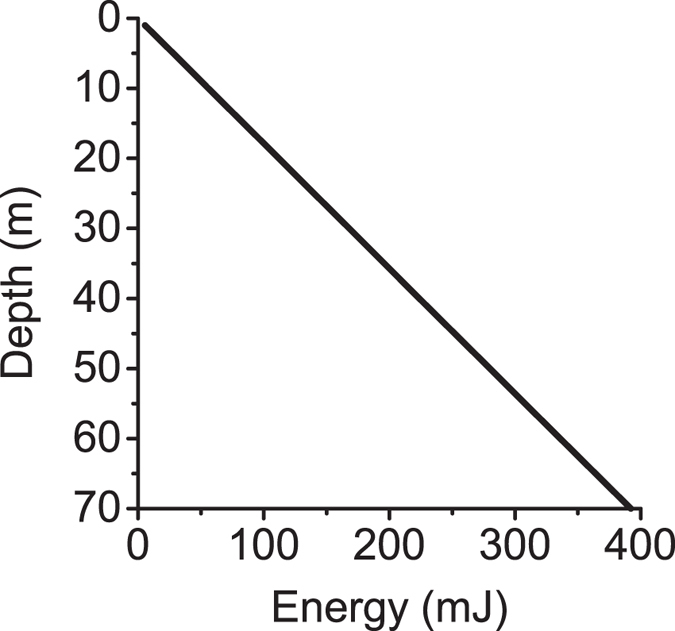
Increase in *Chaoborus* spp. energy expenditure above the resting metabolism (assumed to be 490 mJ d^−1^ at 10 °C) for round-trip migration by active swimming as a function of depth.

Here we present a novel mechanism by which *Chaoborus* spp. use methane-filled gas sacs to aid migration. With this unique adaptation, *Chaoborus* spp. can migrate from sediment at depth with minimal energy expenditure during the night and take advantage of cooler temperatures and absence of predators at depth during the day. Lowered energy demand and effective predator avoidance therefore allow them to substantially expand their habitat and drastically alter the lake ecology. For example, using data from a seven year study of fish biomanipulation in two lakes (Mouse and Ranger Lakes, Dorset, Ontario), Ramcharan *et al*.^37^ found that *Chaoborus* spp. consume more zooplankton than fish, indicating their importance for trophic interactions and energy cycling. Finding the tipping point in the lake’s eutrophication history when methane-fueled migration became feasible (Fig. 1) is therefore an important consideration in paleolimnological research and for understanding corresponding shifts in the lake’s biogeochemistry, greenhouse gas fluxes and food web structure.

## Methods

*Chaoborus* sampling: *Chaoborus* spp. (*C. flavicans*) were collected from Lake Dagow (Stechlin, Germany). Lake Dagow (maximum depth 9.5 m, surface area 0.24 km^2^) is a eutrophic lake with an anoxic hypolimnion in the summer, where *C. flavicans* commonly occur^31^,^38^. Samples were collected in Lake Dagow by deploying a conical net (0.5 m mouth diameter, 200 μm mesh) at about 1–2 m depth from a small boat around midnight when *Chaoborus* spp. migrated to the oxic surface. The animals were kept in surface lake water and immediately transported to the laboratory.

### Sample preparation

In the laboratory, a CH_4_-saturated solution was prepared by bubbling CH_4_ gas into ~20 °C water for ~15 minutes until it reached saturation at ~1.5 mmol L^−1^ 39. *Chaoborus* spp. (4^th^ instars) were sorted for the experiments. These were carefully and individually rinsed (three times) in 5-μm filtered lake water and then placed in the CH_4_-saturated solution for 12 hours (sealed flask) to allow the gas sacs to take up methane from the surrounding water. Afterward the larvae were quickly washed and transferred to the experimental flask (within ~2 minutes).

### Experiments

**Figure 4:**
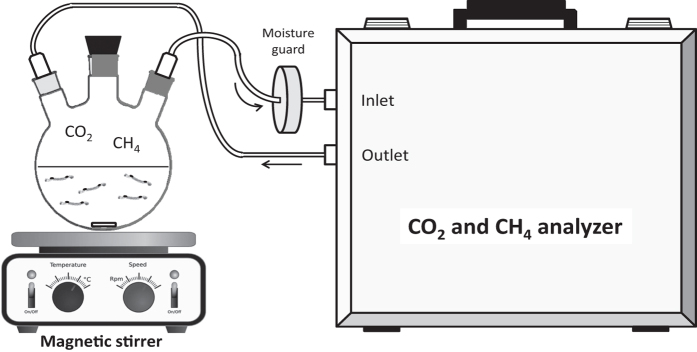
Experimental setup for measuring gas transfer from *Chaoborus flavicans*. *C. flavicans* larvae pre-treated with CH_4_-saturated water were placed in a gas-tight flask connected to gas analyzer in a closed circuit. A magnetic stirrer produced gentle mixing to equilibrate gases between the water phase and the headspace.

To demonstrate methane uptake and redissolution from the larvae, we transferred methane pre-treated larvae to the experimental flask containing 150 ml of filtered lake water. The filtered lake water was at room temperature and at equilibrium with the CH_4_ in the atmosphere (~3 nM CH_4_). The flask was immediately connected to a gas analyzer (CO_2_ and CH_4_; Los Gatos Ultra-Portable Gas Analyzer, Los Gatos Research, California, USA) with a closed circuit. The instrument has a CH_4_ measurement range of 0.01–100 ppm with a repeatability/precision <2 ppb at 1 Hz sampling. A slow-spinning (~60 rpm) magnetic stirrer positioned at the bottom of the flask provided gentle mixing to ensure equal distribution of dissolved CH_4_ (Fig. 4). The expectation was that CH_4_ contained in the larvae’s gas sacs would diffuse across the sac wall and tissues into the surrounding water. Being a sparingly-soluble gas, a large fraction would then pass from the water phase to the headspace, resulting in an increase in headspace CH_4_ concentration over time as a function of abundance of the larvae.

The first experiment was conducted to follow the detailed temporal changes in headspace gas concentrations. Thirty-five fresh, methane pre-treated larvae were transferred to the experimental flask, and changes in headspace gas concentrations were monitored continuously until the CH_4_ concentration reached steady state. We then used 35, 25 and 13 methane treated animals and included a control of 25 larvae pre-incubated in CH_4_-free lake water instead of CH_4_-saturated water to investigate the number of larvae on the total gas exchange. The experiment lasted at least 60 minutes.

### Stoke’s Law

The terminal velocity for the buoyant rise and sink of the larva is calculated from balancing the forces as


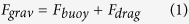


where *F*_*drag*_, *F*_*buoy*_ and *F*_*grav*_ are the drag force, buoyance force, and force due to gravity, respectively. Expanding each of the force terms we get


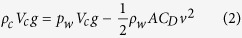


where *A* is the cross-section surface area of the larva (assumed cylindrical), *C*_*D*_ is the drag coefficient, *ρ* is density, *V* is volume, g is gravitational acceleration, and *v* is the terminal velocity. The subscripts *w* and *c* refer to water and *Chaoborus*, respectively. The drag coefficient is a function of the Reynolds number, *Re*, defined as


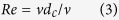


where *d* is the larva’s cross-section diameter, and *ν* is the kinematic viscosity of water. Estimating the initial rising velocity of 5 mm s^−1^ 13 gives *Re* ≈ 4,000. For cylinders with 100 < *Re* < 10^5^, the drag coefficient *C*_*D*_ ≈ 1^40^.

## Additional Information

**How to cite this article**: McGinnis, D. F. *et al*. Porewater methane transport within the gas vesicles of diurnally migrating *Chaoborus* spp.: An energetic advantage. *Sci. Rep.* **7**, 44478; doi: 10.1038/srep44478 (2017).

**Publisher's note:** Springer Nature remains neutral with regard to jurisdictional claims in published maps and institutional affiliations.
